# Exploring morphological and thermal characteristics of chitosan/starch blend films derived from natural sources: incorporating thiourea and urea coupling agents with stearic acid plasticizer

**DOI:** 10.1186/s13065-026-01752-x

**Published:** 2026-03-31

**Authors:** Magd M. Badr, Moataz A. Elsawy, Hamdy M. Naguib, Doaa A. El-Komy, Mahmoud Bekhit, H. A. Elmawgoud

**Affiliations:** 1 Polymer Laboratory, Petrochemicals Department, Egyptian Petroleum Research Institute (EPRI), PO; 11727, Nasr City, Cairo Egypt; 2 Department of Petroleum Applications, Egyptian Petroleum Research Institute (EPRI), 11727, Nasr City, Cairo Egypt; 3 Surfactant Laboratory, Petrochemicals Department, Egyptian Petroleum Research Institute (EPRI), PO; 11727, Nasr City, Cairo Egypt; 4 Processes Design and Development Department, Egyptian Petroleum Research Institute (EPRI), PO; 11727, Nasr City, Cairo Egypt

**Keywords:** Chitosan starch blends, Thiourea, Urea, Coupling agent, Biopolymers, Cast film application

## Abstract

Enhanced biobased polymeric films based on chitosan and starch polysaccharides were prepared and thoroughly investigated. The polymer components were coupled using thiourea and urea as crosslinking agents, with stearic acid incorporated as a plasticizer. The Fourier Transform Infrared spectroscopy (FTIR) and Energy Dispersive X-ray spectroscopy (EDX) data confirmed the successful crosslinking reaction through specific amide peak shifts and elemental distribution. Morphological investigations using Scanning Electron Microscopy (SEM) revealed that urea produced a homogeneous matrix, whereas thiourea induced a rougher morphology due to agglomeration. X-ray Diffraction (XRD) analysis revealed that thiourea-modified films achieved a significantly higher crystallinity index (43.2%) compared to urea-modified films (28.9%). Thermal behavior was analyzed using Differential Scanning Calorimetry (DSC), Thermogravimetric Analysis (TGA), and Differential Thermal Analysis (DTA), demonstrating that thiourea composites exhibited superior thermal stability with a residual weight of ~ 40% at 600 °C. Kinetic parameters were calculated using the Kissinger-Akahira-Sunose (KAS) model, confirming the enhanced activation energy of the crosslinked networks. The synthesized biobased polymers demonstrated thermal and mechanical stability suitable for applications in agriculture and packaging.

## Introduction

There is a growing interest in developing biodegradable materials derived from natural polymers, driven by the anticipated depletion of fossil fuels and the environmental challenges associated with conventional packaging practices. Substances like starch, cellulose, and polysaccharides such as chitin (including its derivative, chitosan) are considered vital raw materials for future biorefineries due to their widespread availability [1].

Starch, in particular, stands out as a highly utilized raw material for crafting biodegradable films, given its accessibility, cost-effectiveness, renewability, biocompatibility, and non-toxic nature [2]. Moreover, starch can be easily converted into a thermoplastic substance known as thermoplastic starch (TPS) by disrupting interactions between polymeric chains under specific temperature and mechanical energy conditions, facilitated by a plasticizer such as water or glycerol [3].

Combining starch with other biopolymers and substances, particularly those with antioxidant and antimicrobial properties, has been proposed to enhance both the physical and functional attributes of starch films, which is crucial in applications such as functional food packaging. Numerous studies have focused on developing and characterizing films made from starch and chitosan due to their significant potential in packaging and food preservation technology [4–7]. The notable antibacterial properties of chitosan against a broad spectrum of pathogenic and spoilage microorganisms, including Gram-negative and Gram-positive bacteria and fungi, contribute to its popularity in these applications [8–10]. However, many studies on starch-chitosan films involve the time-consuming process of pre-gelatinizing starch before adding chitosan and casting. While straightforward, this method is impractical for large-scale industrial production.

Recent investigations have explored the influence of temperature and screw speed on the mechanical and barrier properties of TPS. TPS can be produced using conventional melt-mixing or extrusion machinery, offering an alternative to the time-intensive approach of pre-gelatinization [11, 12]. Some studies have also evaluated the transparency and films made of starch, chitosan, and oregano oil. Chitosan, with its exceptional antibacterial and film-forming capabilities, can be employed to create antimicrobial films [13].

Chitosan, owing to its remarkable biodegradability, bioactivity, and biocompatibility, has emerged as a focal point in the realm of biomaterials [14, 15]. Its versatility extends to the pharmaceutical, medical, and cosmetic industries, where it interacts effectively with elements of the skin and hair carrying negative electrical charges [16]. Widely utilized in mouthwashes, dental pastes, hair gels, shampoos, and as a cationic film-forming ingredient in skincare products [17], chitosan also finds applications in food packaging and coating [18, 19].

The interaction between chitosan’s positively charged amino (–NH3+) groups and the negatively charged carboxylate (–COO–) groups on bacterial cell walls underlies its bacteriostatic and bactericidal effects [9, 20]. These antibacterial properties may result from membrane rupture and protein leakage from cells [21]. The film-forming ability and antibacterial qualities of chitosan make it particularly valuable in food packaging. In the context of raw food or food products, films incorporating chitosan can limit moisture loss and reduce dripping from meats and fish. Additionally, the antioxidant and antibacterial coating of chitosan films can prevent flavor loss and the absorption of foreign odors [22, 23].

Both starch and chitosan are considered excellent materials for food packaging due to their biodegradable, biocompatible, affordable, hydrophilic, and non-toxic characteristics [24]. The synergistic properties of starch and chitosan have garnered significant interest not only in food additives and packaging but also in biotechnology, biomedicine, and cosmetics [25, 26]. In applications such as drug delivery and tissue engineering, chitosan and starch hydrogels are frequently employed [27]. While the direct use of native starch for hydrogels is impractical due to its poor processability, dimensional stability, and mechanical properties for end products [28]. However blending chitosan with other polysaccharides or utilizing cross-linking agents can enhance the mechanical characteristics of starch/chitosan films [29]. Creating blends by combining two or more polymers is a proven method for achieving specific physical qualities and ensuring compatibility. Considerable research has explored starch composite films with various polymers, and films composed of chitosan and starch have been recommended for use in food packaging and medical applications [27]. The combination of starch and chitosan contributes to mechanical stability and reduced crystallinity, with polysaccharides offering biocompatibility and biodegradability [29]. Scientists have made several efforts to prepare several types of homogenous polymer blends and modification [30–36].

In this study, we assess the viability of thiourea and urea, both cost-effective and readily available materials, as crosslinking agents to facilitate the combination of chitosan and starch polymers in forming biopolymer films. Literature indicates that both agents effectively modify polymer networks; however, they possess distinct safety profiles. While urea is generally recognized as safe and widely used, thiourea is significantly more reactive and entails potential health risks due to its toxicity. Therefore, this study not only evaluates their crosslinking efficiency but also implicitly considers the trade-off between performance enhancement and the safety constraints required for potential food packaging applications. Our focus is on studying the impact of the thiourea crosslinking agent on the chemical structure and exploring the morphological, thermal, chemical, and physical properties through various analytical techniques, including FTIR, EDX, SEM, XRD, DMA, DSC, TGA, and DTA. The thermal data obtained were used to determine kinetic parameters employing methods such as Coats-Redfern, Friedman, Flynn-Wall-Ozawa (FWO), and Kissinger-Akahira-Sunose (KAS).

## Experimental materials and methods

### Materials

Chitosan with a deacetylation degree of 92% and a molecular weight of 134 kDa was sourced from ChitoFarma Fertilizers Company, located at New Salhia City, Egypt. The chitosan used in this study is known for its high purity and biocompatibility. Food-grade corn starch was obtained from the local market and served as the primary polysaccharide component in the biobased films. It was selected for its availability and suitability in forming biodegradable matrices with chitosan. Thiourea (99% purity) and urea (99.5% purity), utilized as crosslinking agents, were purchased from El-Nasr Pharmaceutical Company of Chemicals, Egypt. These chemicals were chosen for their cost-effectiveness and ability to facilitate the crosslinking between chitosan and starch, essential for enhancing the mechanical and thermal properties of the films. Stearic acid (97% purity), obtained from the same supplier, was employed as a plasticizer to improve the flexibility and processability of the biopolymer films. No further modifications were made to any of the ingredients prior to their use in film preparation. All materials were used as received to maintain consistency and to ensure that any observed effects could be attributed directly to the formulations and processing methods employed in this study.

### Preparation of the thiourea and urea/chitosan/starch polymer blend samples

**Scheme 1:**
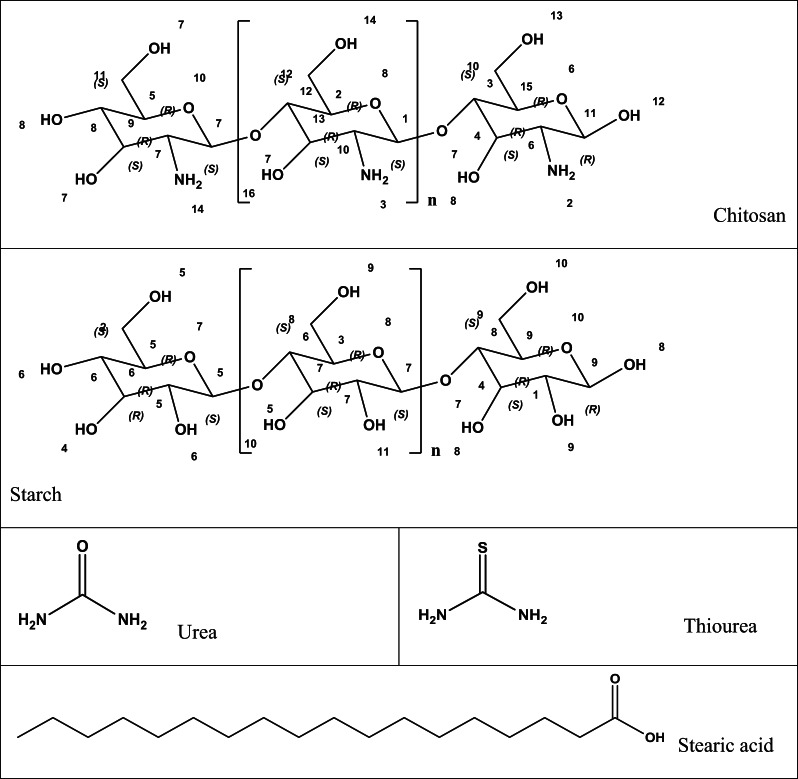
The chemical structure of the formulation ingredients

**Scheme 2:**
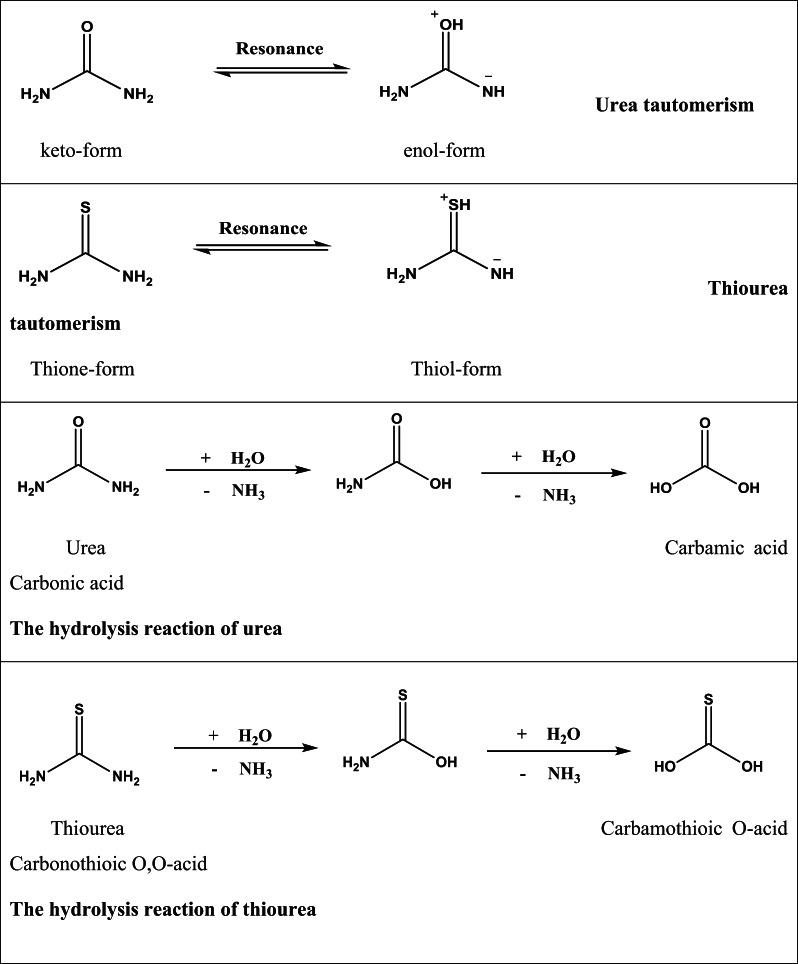
Urea and thiourea isomerization and hydrolysis

4 g of chitosan (Chi) were dissolved in 200 ml of 2% aqueous acetic acid solution. This solution was added dropwise to a stirred mixture of 4 g of starch (Str) in 200 ml of distilled water, which had been brought to a boil and stirred for 10 min. Subsequently, 1 gram of either thiourea or urea was added dropwise, and the mixture was stirred continuously for 1 h to produce the ThU/Chi/Str and Ure/Chi/Str samples. For film formation, 0.3 ml of stearic acid (Str) was added to each solution 30 min before the stirring process was completed. The resulting material samples were then cast onto Petri dishes and dried overnight in an oven at 40 °C. To ensure statistical reliability, three independent replicates were prepared for each blend formulation (Chi/Str, Chi/Str/Urea, and Chi/Str/Thio), and the reported characterization results represent the average of these measurements (Schemes 1 and 2).

## Results and discussion

The coupling agent of thiourea and urea is known for its ability to react in two steps with both the hydroxyl groups and the amino groups. During the first step, thiourea and urea react with hydroxyl groups, followed by a second step where they react with amino groups, forming a crosslinked structure. For this reason, thiourea and urea were selected to be used for the chemical curing of the two polymeric materials. Both starch and chitosan cast film formations in previous studies were reported to suffer from poor film appearance and unsatisfactory mechanical properties [2, 28]. In the current study stearic acid was used as a plasticizer for the thiourea and urea chitosan/starch blend while glycerol was reported as a plasticizer for chitosan/starch blends for film formation [37–39]. Stearic acid is one of the common polymer plasticizers [40]. The stearic acid also plays a reactive role in the polymer matrix due to its terminal carboxylic group, which can interact with residual hydroxyl groups and unreacted thiourea or urea molecules, helping to terminate the polymerization process and stabilize the final structure.

Importantly, stearic acid was not added at the beginning of the reaction. Instead, it was introduced after the initial crosslinking steps to allow sufficient time for the propagation of the thiourea/urea-mediated crosslinking reactions. This controlled addition helped optimize the crosslinking density and the final film properties.

### Characterization

#### FTIR analysis of the prepared samples

**Fig. 1:**
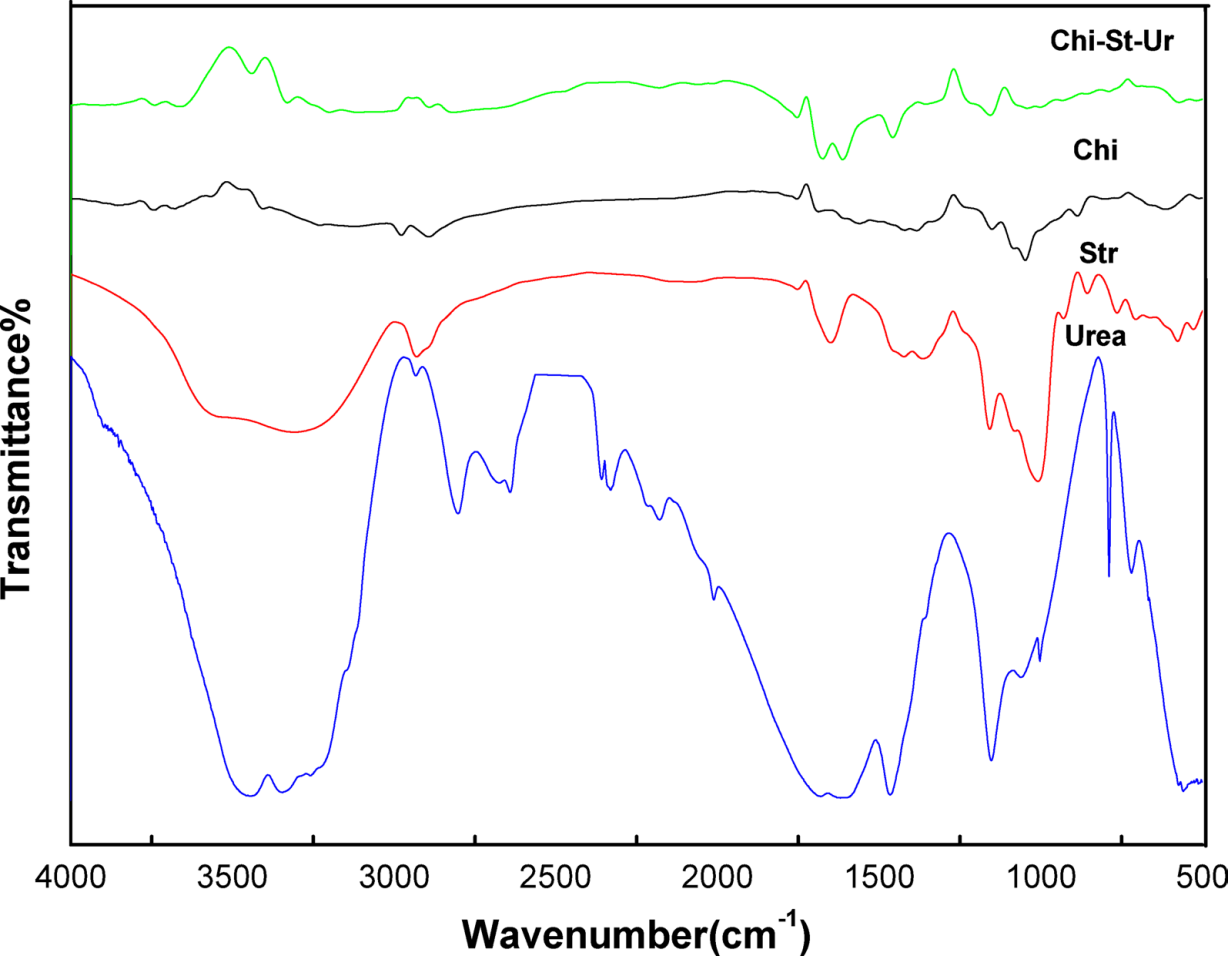
IR spectrum of chitosan (Chi) and chitosan/starch (Chi/Str) blend films with urea (Chi/Str/Urea) as a coupling agent

**Fig. 2:**
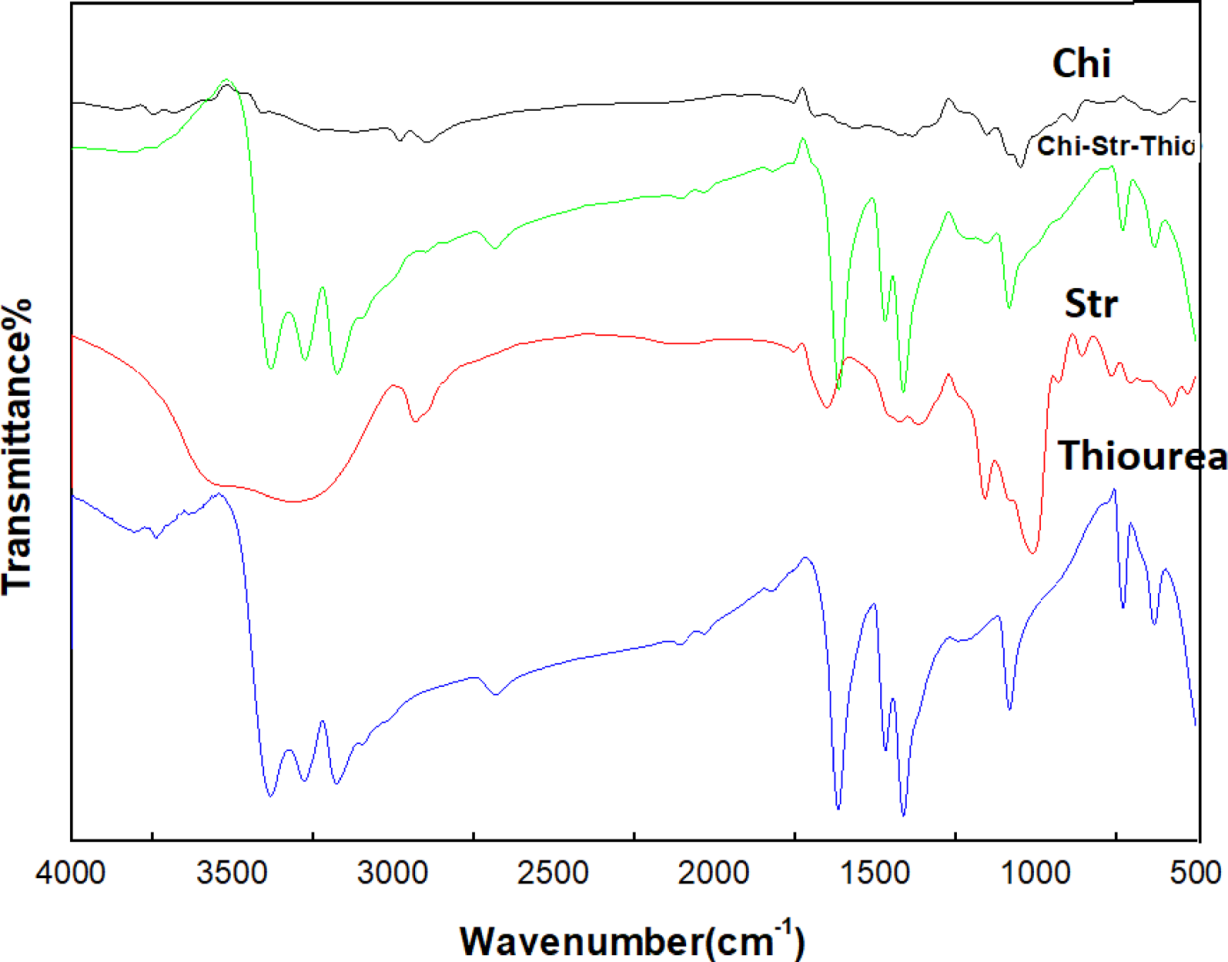
IR spectrum of chitosan (Chi) and chitosan/starch (Chi/Str) blend films with thiourea (Chi/Str/Thio) as a coupling agent

The IR spectrum in Fig. 1 illustrates the transmittance (%) versus wavenumber (cm^− 1^) for various chitosan (Chi) and starch (Str) blend films, focusing on the incorporation of urea as a coupling agent. The spectrum of pure chitosan (Chi) exhibits characteristic bands such as O-H and N-H stretching around 3000–3500 cm^− 1^, indicative of hydroxyl and amino groups, and C-H stretching vibrations at 2870 and 2920 cm^− 1^ [41]. The amide I band at 1650 cm^− 1^ and amide II band at 1550 cm^− 1^ correspond to C = O stretching and N-H bending, respectively. Additionally, the peaks in the range of 1150–1050 cm^− 1^ are attributed to C-O-C stretching vibrations, reflecting the polysaccharide structure. In the chitosan/starch blend (Chi/Str) film spectrum, enhanced O-H and N-H stretching suggests stronger interactions between chitosan and starch. The retention of amide I and II bands confirms the preservation of chitosan’s structural features, while C-O-C stretching indicates the polysaccharide nature of the blend. The incorporation of urea (Chi/Str/Urea) results in broadened O-H and N-H stretching bands, indicating enhanced interactions. The presence of C = O stretching around 1670–1630 cm^− 1^ confirms urea incorporation, with amide band modifications further demonstrating urea’s influence on the matrix. Specifically, the Amide I band shifted from 1650 cm^− 1^ (pure chitosan) to 1635 cm^− 1^, and the Amide II band shifted from 1550 cm^− 1^ to 1542 cm^− 1^, confirming the participation of amino groups in the crosslinking reaction. Structural changes are also indicated by altered peaks in the 1200–1000 cm^− 1^ region. The addition of stearic acid as a plasticizer is evident from the characteristic C = O stretching band at 1730 cm^− 1^, which is more prominent in the spectra of the urea-coupled films, suggesting better dispersion facilitated by the coupling agent. Enhanced C-H stretching vibrations around 2900–2800 cm^− 1^ also reflect the presence of stearic acid. In Fig. 2, the IR spectrum for chitosan/starch blend films with thiourea (Chi/Str/Thio) shows broader O-H and N-H stretching bands, indicating stronger interactions within the matrix. New peaks around 2100–2200 cm^− 1^, corresponding to N = C=S stretching, confirm thiourea integration. Shifts in the amide I band (to 1628 cm^− 1^) and amide II band (to 1538 cm^− 1^) reflect the interaction between thiourea and the polymer matrix. Furthermore, the broadening and shift of the -OH stretching peak from 3450 cm^− 1^ to 3420 cm^− 1^ indicate increased hydrogen bonding density. Additional peaks in the 1200–1000 cm^− 1^ region suggest structural changes due to thiourea. Similar to the urea-coupled films, the presence of stearic acid is evidenced by the characteristic C = O stretching band at 1730 cm^− 1^, which is more prominent in the spectra of the thiourea-coupled films, indicating better dispersion facilitated by the coupling agent. Enhanced C-H stretching vibrations around 2900–2800 cm^− 1^ also reflect the presence of stearic acid. Overall, the IR spectra reveal significant molecular interactions and structural modifications in the chitosan/starch blend films due to the incorporation of urea and thiourea coupling agents and the stearic acid plasticizer. These findings are crucial for understanding the films’ morphological and thermal behaviors, guiding their potential applications [42].

### Elemental analysis using EDX-mapping of chitosan/starch blend modified with thiourea and urea

**Fig. 3:**
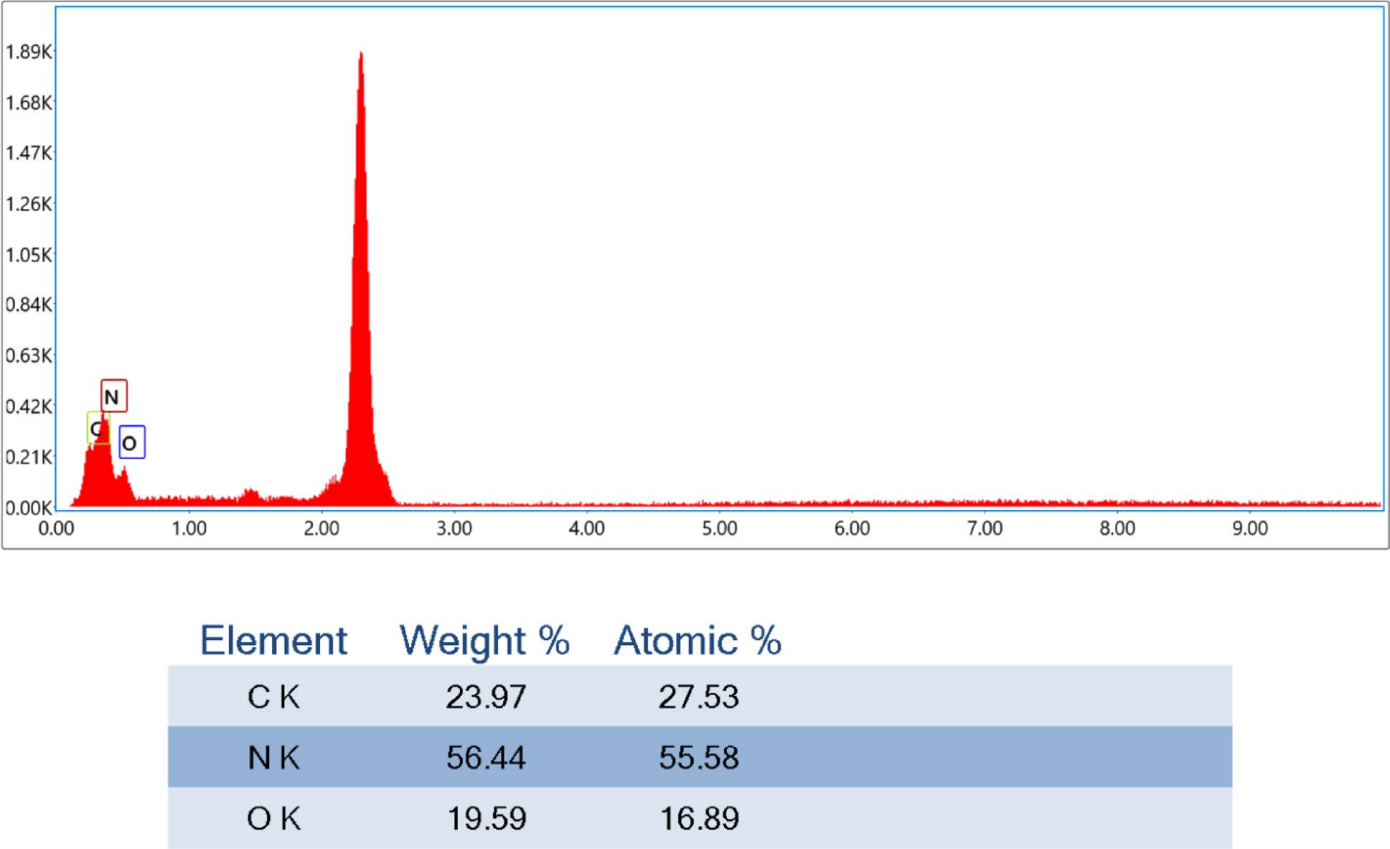
EDX-Mapping of ThU/Chi/Str. (Values represent the mean of three replicates with a standard deviation of ± 1.5%)

**Fig. 4:**
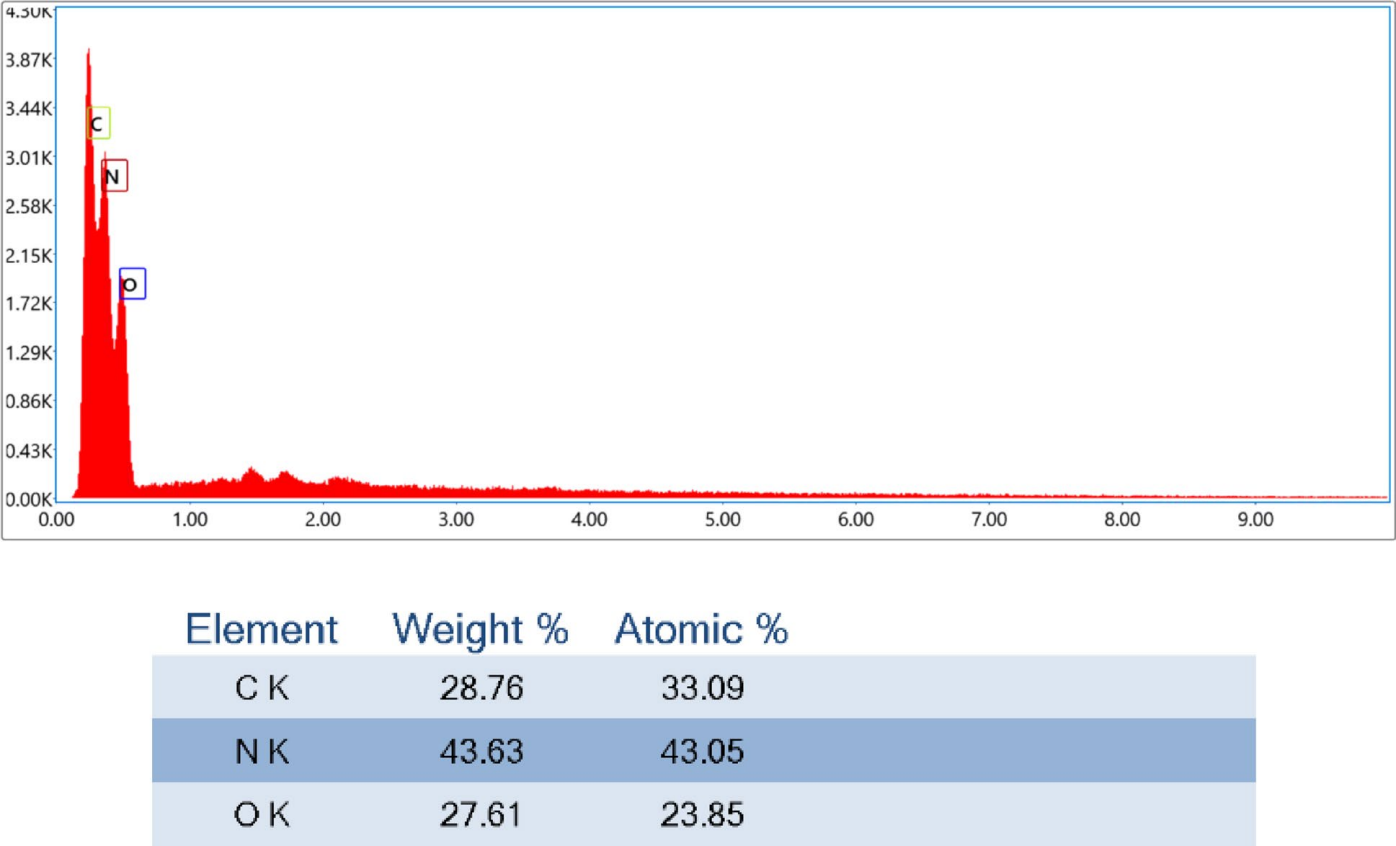
EDX-Mapping of Ure/Chi/Str. (Values represent the mean of three replicates with a standard deviation of ± 1.8%)

Figures 3 and 4 indicate the EDX-mapping of Chi/Str/Urea and Chi/Str/Thio blends, respectively. The related percentages provide the incorporation of reactants with different compatibility observations. Although the differentiation presents in the intensities, both figures reflect the successful compounding by the effect of coupling agents, which alter the elemental analysis. The plasticizing effect may promote the overall characteristics, as shown after. The mapping figures confirm the microstructural change in the formed blends.

### X-ray diffraction

**Fig. 5:**
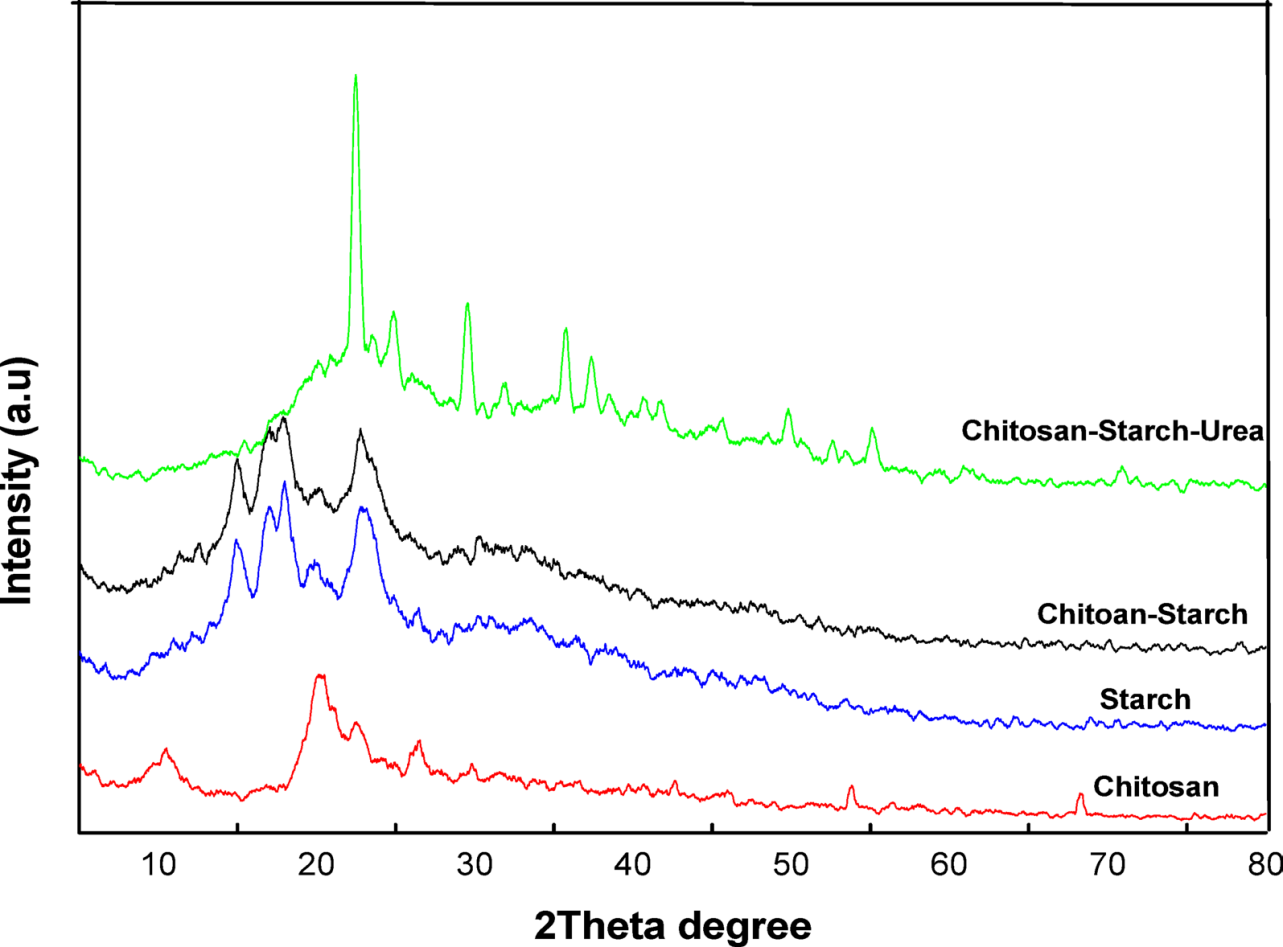
X-ray diffraction (XRD) patterns of chitosan (Chi), starch (Str), chitosan/starch (Chi/Str) blend, and urea-incorporated chitosan/starch (Chi/Str/Urea) blend films, showing changes in crystalline structures

**Fig. 6:**
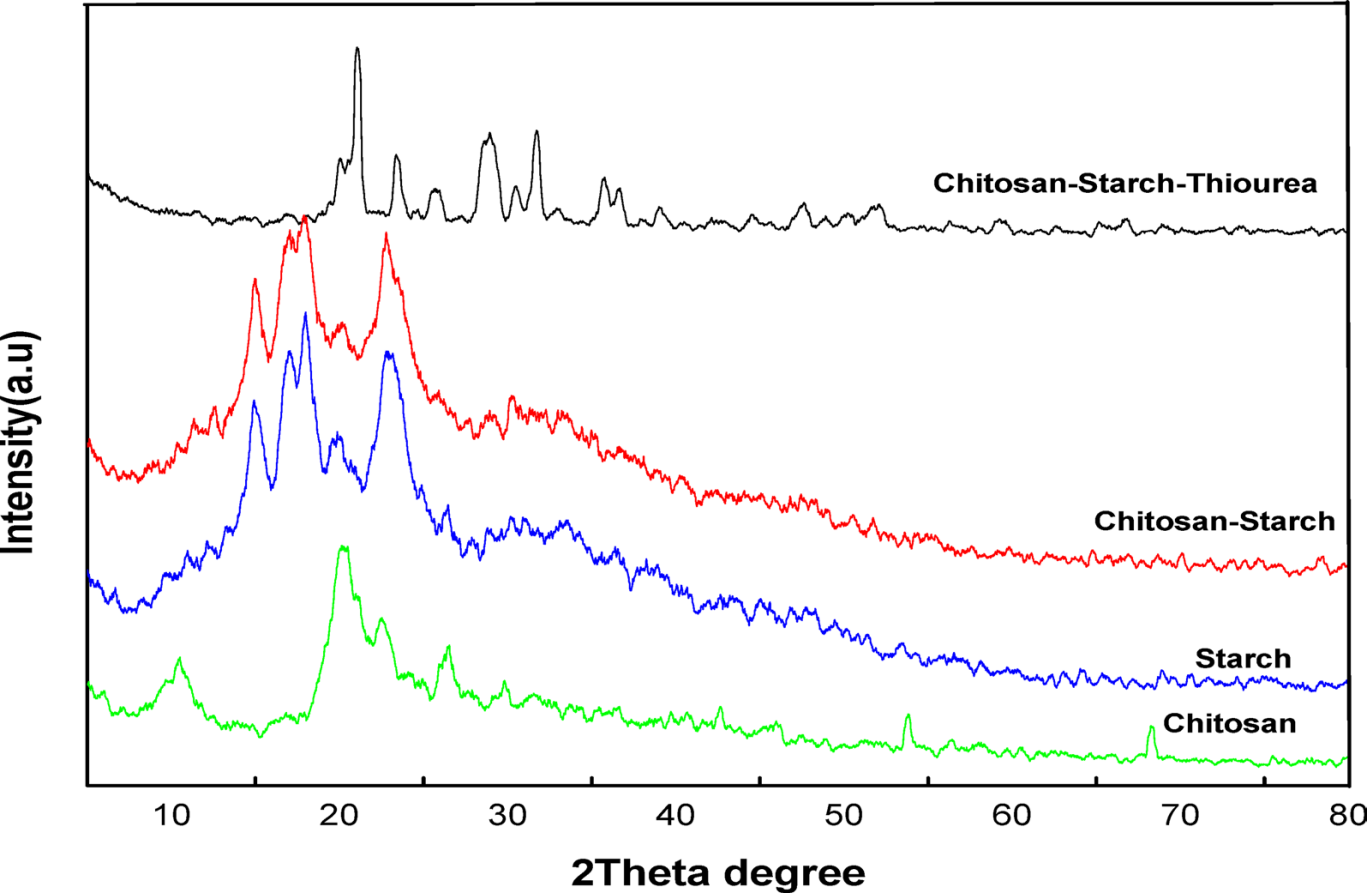
X-ray diffraction (XRD) patterns of chitosan (Chi), starch (Str), chitosan/starch (Chi/Str) blend, and thiourea-incorporated chitosan/starch (Chi/Str/Thio) blend films, indicating increased crystallinity and formation of a new crystalline phase

The X-ray diffraction (XRD) patterns in the Figs. 5 and 6 provide insights into the crystalline structures of chitosan (Chi), starch (Str), chitosan/starch blends (Chi/Str), and the blends incorporated with urea (Chi/Str/Urea) and thiourea (Chi/Str/Thio). The chitosan/starch blend was prepared at a 50:50 (wt%/wt%) ratio.

In Fig. 5, the XRD spectrum of pure chitosan (Chi) displays broad peaks around 2θ = 10° and 20°, indicating its semi-crystalline nature. Starch (Str) exhibits characteristic peaks at around 2θ = 17°, 20°, and 23°, reflecting its crystalline structure. In the chitosan/starch blend (Chi/Str), the XRD pattern shows a combination of diffraction peaks from both chitosan and starch, with a noticeable reduction in peak intensity. The characteristic peak of chitosan at 2θ = 20° becomes overlapped with starch peaks, making it less visible. This suggests partial disruption of the crystalline structure of starch due to interaction with chitosan, leading to a more amorphous blend. The broadening of peaks around 2θ = 10° and 20° indicates increased amorphous content and better intermixing between chitosan and starch.

The XRD pattern of the urea-incorporated chitosan/starch blend (Chi/Str/Urea) shows significant changes compared to the Chi/Str blend. The characteristic peaks of starch become less prominent, while new peaks appear around 2θ = 12° and 22°, indicating the formation of a new crystalline phase. This suggests that urea acts as a coupling agent, enhancing the interactions between chitosan and starch, and promoting the formation of a more ordered structure. The overall reduction in peak intensity and the appearance of new peaks indicate a complex interplay between chitosan, starch, and urea, resulting in a modified crystalline structure. The crosslink density in the matrix influences the crystallinity, where increased crosslinking leads to reduced molecular mobility and changes in crystal domain arrangement.

In Fig. 6, the XRD pattern for chitosan/starch blend films with thiourea (Chi/Str/Thio) shows even more pronounced changes. The broad peaks around 2θ = 10° and 20° become sharper and more intense, indicating increased crystallinity due to the integration of thiourea. New diffraction peaks around 2θ = 15° and 25° further confirm the formation of a distinct crystalline phase, suggesting stronger interactions within the polymer matrix. The combination of chitosan, starch, and thiourea results in a well-ordered structure, as evidenced by the sharper and more defined peaks compared to the urea-incorporated blend. To quantitatively assess the impact of crosslinking on the film structure, the Crystallinity Index (CrI %) was calculated using the Segal empirical method. The pure chitosan/starch blend showed a relatively low crystallinity index of 18.5%. Upon crosslinking, the urea-modified blend exhibited an increased CrI of approximately 28.9%, whereas the thiourea-modified blend showed the highest CrI of 43.2%. This progressive increase (Pure < Urea < Thiourea) confirms that the crosslinkers, particularly thiourea, facilitate a more ordered packing of the polymer chains, likely due to the enhanced hydrogen bonding and crosslinking capability of the sulfur group compared to the oxygen in urea. This supports the observation of sharper diffraction peaks in the XRD patterns.These XRD findings, combined with the IR results; highlight the significant impact of thiourea and urea coupling agents on the structural properties of chitosan/starch blend films, guiding their potential applications.

### Morphological characterization using SEM analysis of ThU/Chi/Str and Ure/Chi/Str

Regarding the control unmodified blend, previous studies [2, 39] have extensively characterized pure chitosan/starch films, reporting a discontinuous morphology with visible micro-phase separation due to the lack of covalent crosslinking. In comparison to this established baseline, the SEM images of the chitosan/starch/urea (Chi/Str/Urea) and chitosan/starch/thiourea (Chi/Str/Thio) blend films provide valuable insights into their morphological characteristics, revealing significant differences in their microstructures due to the incorporation of urea and thiourea as coupling agents.

**Fig. 7:**
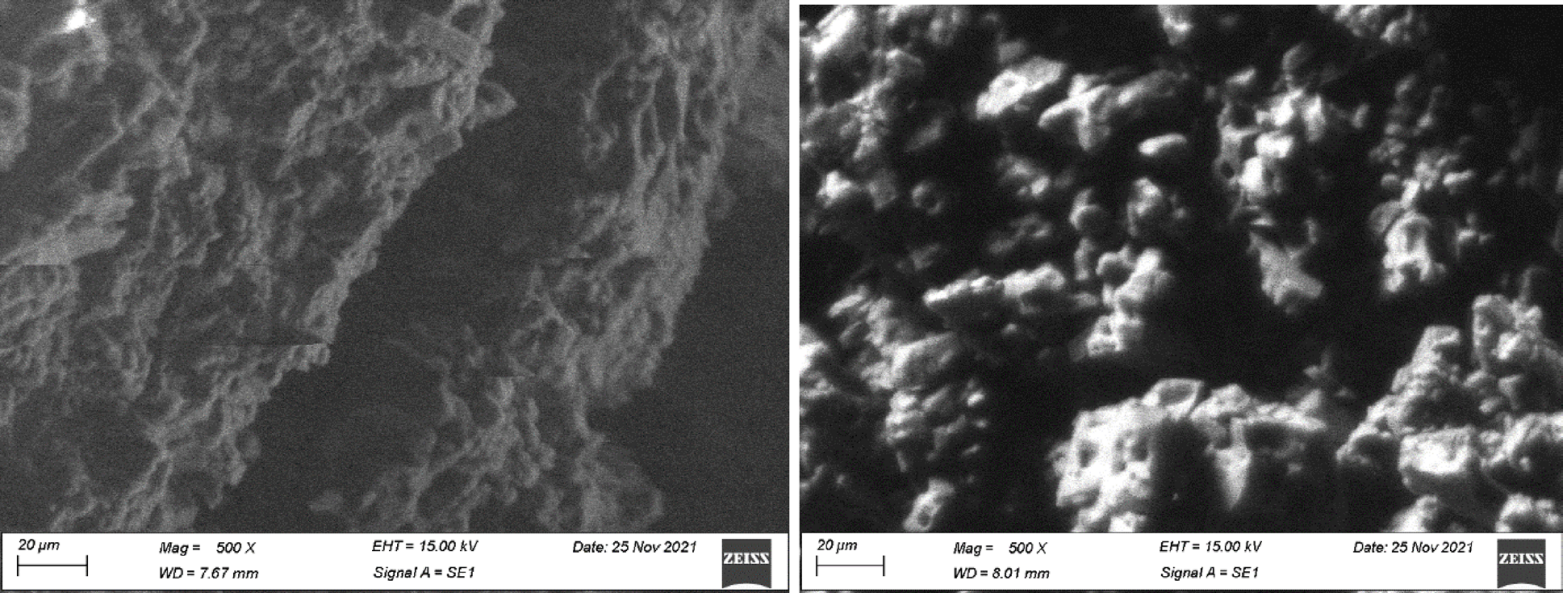
SEM image of chitosan/starch/thiourea (Chi/Str/Thio) blend film, showing partial compatibility with noticeable particle coagulation and grain formation

The SEM image of the Chi/Str/Thio blend (Fig. 7) shows a surface with partial compatibility and noticeable particle coagulation, forming distinct grains. The surface morphology appears irregular and rough, indicating areas where the polymer components are not fully integrated. The presence of these aggregated particles suggests that while thiourea promotes some degree of interaction between chitosan and starch, it does not completely facilitate a uniform blending at the microscopic level. This partial compatibility could be due to the specific interactions of thiourea with the polymer chains, which may enhance crystallinity but also lead to phase separation and particle formation. It is important to harmonize this morphological observation with the XRD data. While thiourea induced high crystallinity (43.2%), this strong self-association and molecular ordering of the polymer chains led to the formation of distinct crystalline aggregates. These aggregates result in the rougher, less homogeneous surface morphology observed here, unlike the urea blends where lower crystallinity allowed for smoother, albeit less thermally stable, surface integration.

**Fig. 8:**
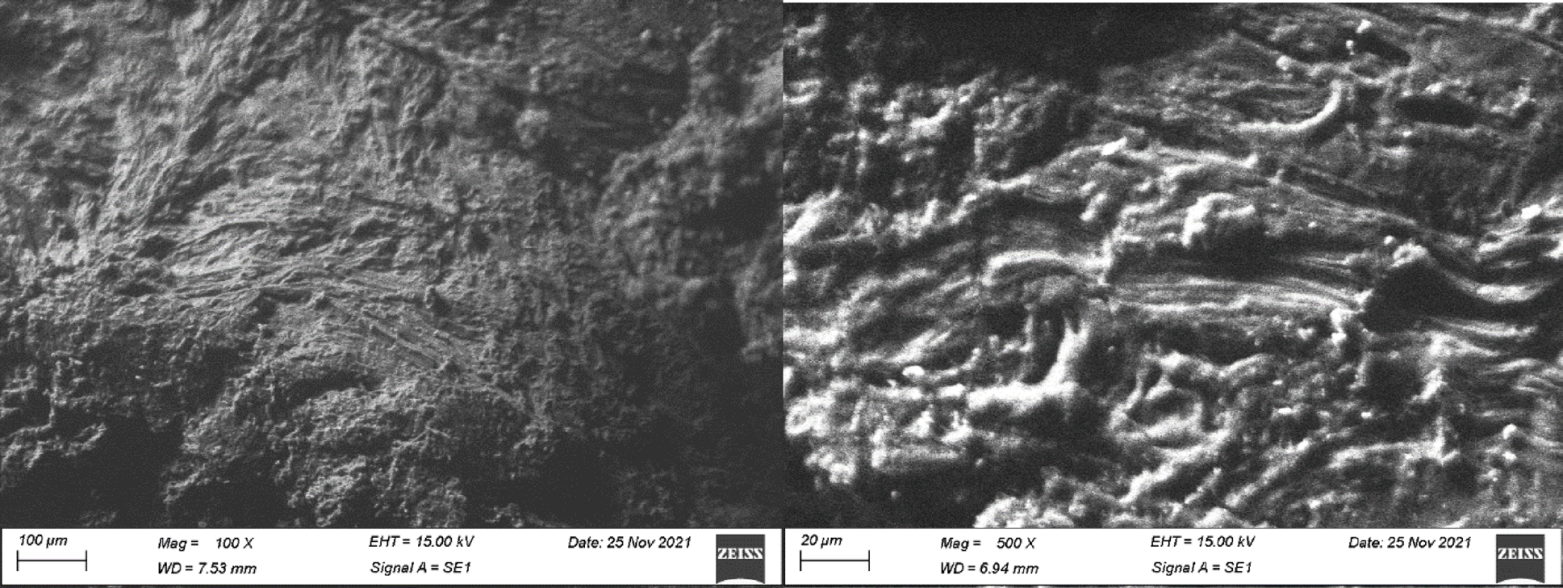
SEM image of chitosan/starch/urea (Chi/Str/Urea) blend film, indicating good compatibility with a homogeneous and continuous matrix phase

In contrast, the SEM images of the Chi/Str/Urea blend (Fig. 8) exhibits a much more homogeneous and continuous matrix. The polymer chains of chitosan and starch appear to form a cohesive phase without noticeable particle coagulation. This uniform matrix indicates good compatibility between the components, facilitated by the urea coupling agent. The smooth and continuous surface morphology suggests that urea effectively enhances the interaction between chitosan and starch, leading to a well-integrated blend. The absence of distinct particles and the even distribution of the polymer phases imply that urea acts as a better compatibilizer compared to thiourea, promoting a more uniform and compatible composite material.

These observations are consistent with the XRD and IR results, which showed distinct differences in the crystalline and chemical structures of the blends with urea and thiourea. The urea-incorporated blend exhibited better compatibility and more homogeneous mixing, as evidenced by the smooth and continuous surface in the SEM image. In contrast, the thiourea-incorporated blend displayed increased crystallinity and particle formation, indicating partial compatibility and phase separation.

### Thermogravimetric analysis

**Fig. 9:**
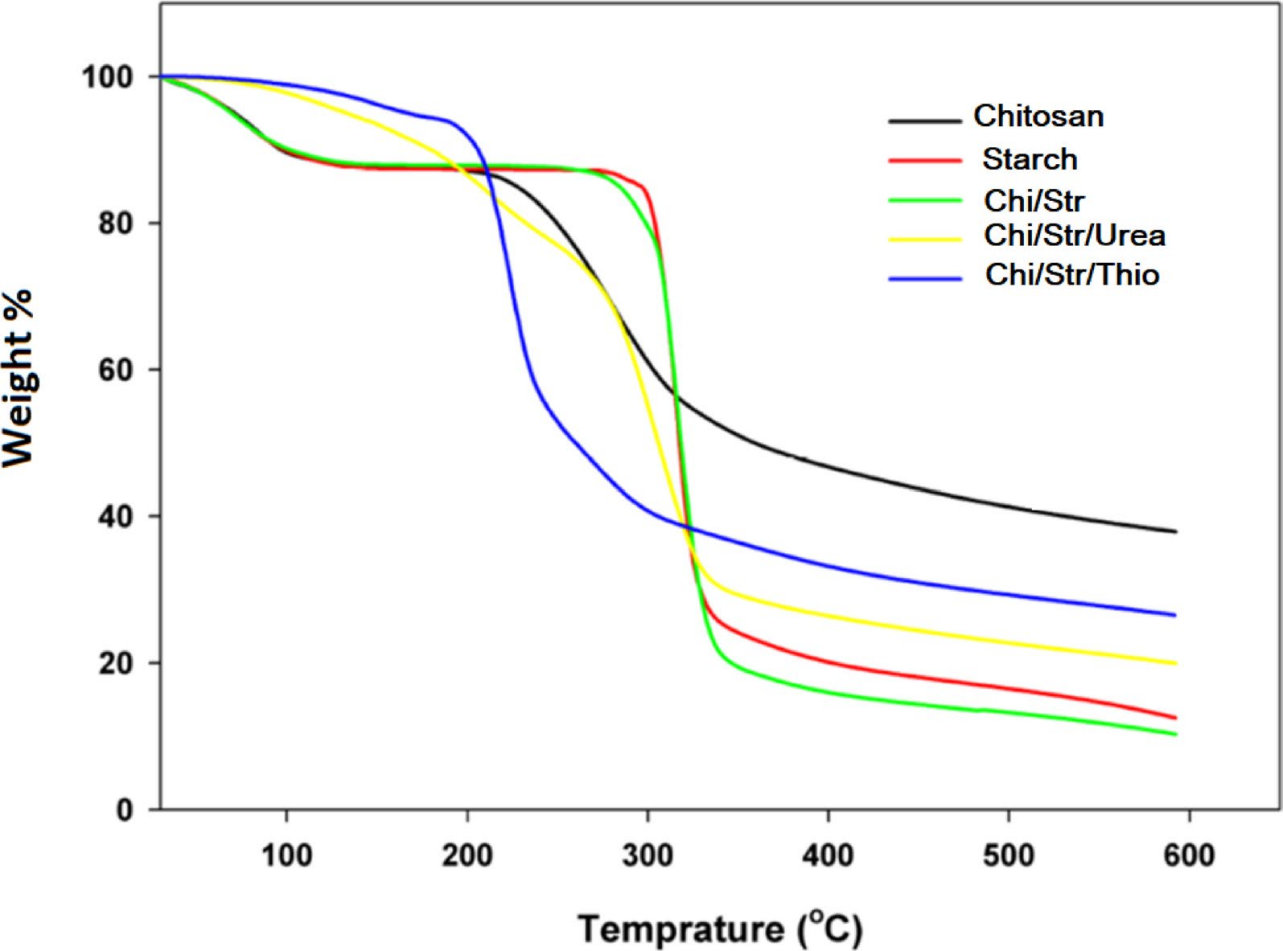
Thermogravimetric Analysis (TGA) curves of pure chitosan, starch, and their composites with and without coupling agents (urea and thiourea)

Thermogravimetric Analysis (TGA) was employed to scrutinize the thermal stability and decomposition characteristics of chitosan, starch, and their composite materials, including Chi/Str, Chi/Str/Urea, and Chi/Str/Thio. Figure 9 showcases the TGA curves, delineating weight loss against temperature for each specimen under a nitrogen atmosphere. The TGA profile of chitosan displays an initial minor weight loss (~ 10%) below 100 °C, attributable to moisture evaporation, with the major weight loss commencing around 230 °C and persisting until approximately 350 °C, signaling the breakdown of the chitosan polymer backbone. This primary decomposition phase accounts for a substantial weight loss, diminishing the weight to roughly 30% of the initial value, with a final residue of approximately 40% at 600 °C, potentially owing to the presence of thermally stable char. The TGA curve for starch mirrors a similar initial moisture loss below 100 °C, with a principal thermal degradation unfolding between 290 °C and 350 °C, hinting at slightly superior thermal stability, compared to chitosan, accompanied by a significant weight loss leaving about 18% residual weight at 600 °C. The TGA curve representing the Chi/Str composite showcases an initial weight loss attributed to moisture evaporation below 100 °C, with the primary degradation phase occurring between 280 °C and 350 °C, augmented thermal stability compared to the pristine specimen, suggesting a synergistic effect between chitosan and starch in enhancing the composite’s thermal stability. The TGA curve for the Chi/Str/Urea composite delineates a slightly distinct thermal degradation profile, with moisture loss initiating below 100 °C, followed by a two-step degradation process spanning from 200 °C to 300 °C (with a maximum degradation rate, T_max1_, at 285 °C), and a secondary phase extending to 400 °C (T_max2_ at 345 °C), potentially attributable to additional components or interactions affecting its thermal resistance, with a residual weight of approximately 23%. The TGA curve for the Chi/Str/Thio composite portrays minimal initial moisture loss below 100 °C, and succeeded by a major weight loss starting around 200 °C and persisting until 400 °C (T_max_ at 310 °C), boasting the highest thermal stability among composites, exhibiting a residual weight of approximately 32% at 600 °C, indicating that thermal treatment or modifications in the Chi/Str/Thio composite contribute to a more thermally stable structure at higher temperatures, likely attributed to enhanced cross-linking or the presence of thermally resistant additives.

### Dynamic mechanical analysis

**Fig. 10:**
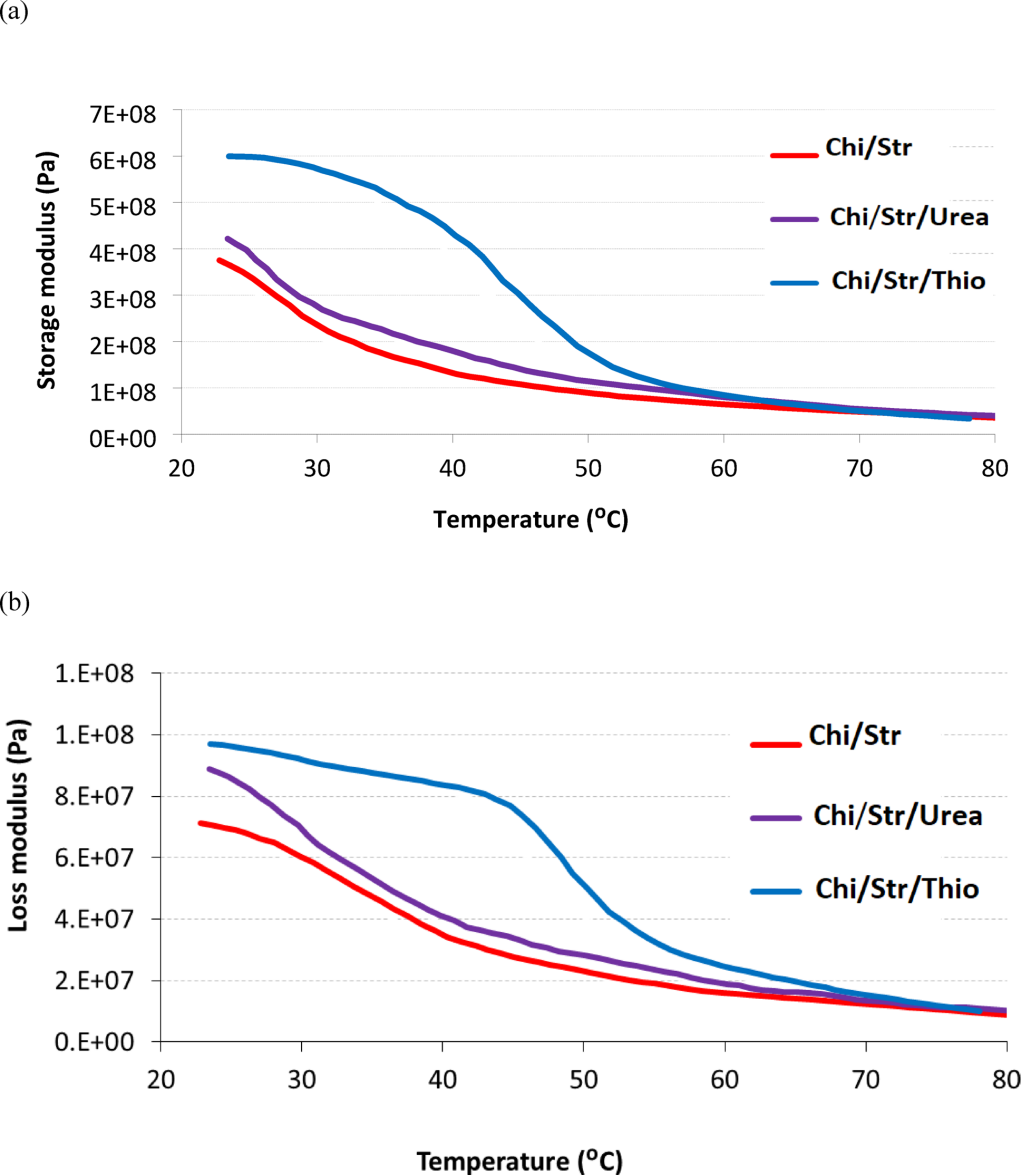
**a**: Storage modulus versus temperature for chitosan/starch (Chi/Str), chitosan/starch/urea (Chi/Str/Urea), and chitosan/starch/thiourea (Chi/Str/Thio) composites, showing viscoelastic stability before the glassy zone and higher storage moduli for the urea and thiourea composites. **b**: Loss modulus versus temperature for chitosan/starch (Chi/Str), chitosan/starch/urea (Chi/Str/Urea), and chitosan/starch/thiourea (Chi/Str/Thio) composites, indicating higher loss moduli for the urea and thiourea composites and improved stability for the thiourea composite at elevated temperatures

The results of DMA are represented in Fig. 10a, b, respectively, indicating the values of storage and loss moduli for Chi/Str, Chi/Str/Urea, and Chi/Str/Thio composites. All values decrease dramatically with increasing testing temperature, as usual for thermoplastics. Generally, it was reported that starch-based composites have an effective filling mechanism due to plasticization and stiffness [43]. In Fig. 10a, all composites show viscoelastic stability before the glassy zone due to their stability. Both the Chi/Str/Urea and Chi/Str/Thio composites have a higher storage modulus than the Chi/Str composite. This originates from the restricted mobility of the blend segments, and the chemical attachment in their structures. The maximum modulus (6 × 10^8^ Pa) was recorded by the Chi/Str/Thio composites, where the enhancement percentages are about 56% and 43% related to Chi/Str/Urea and Chi/Str, respectively. A similar behavior is observed in the characterized loss modulus, as plotted in Fig. 10b. The measured values are 7 × 10^7^ Pa, 8.9 × 10^7^ Pa, and 9.6 × 10^7^ Pa for the Chi/Str, Chi/Str/Urea, and Chi/Str/Thio composites, respectively. It is expected that the sulphur atom in thiourea able to support the composite with a crosslinked network; consequently, the mechanical properties are enhanced. The storage and loss moduli show lower values at elevated temperatures due to the free motion of the composite segments. However, the Chi/Str/Thio shows a more stable modulus, especially with the loss plateau, as seen in Fig. 10b.

Overall, with both moduli, the Chi/Str/Thio has the enhanced character regarding the improved modulus at higher temperatures, and the effective plasticizing agent.

### Kinetic analysis

A great deal of research has been done on the kinetics of starch’s heat breakdown. A number of techniques have been created and assessed to explain and forecast the kinetics of starch’s heat breakdown. Among those techniques, model-free techniques have shown effective in reproducing the actual data in a multi-step kinetics simulation. By employing these techniques, the process can be identified as activation energy Ea dependent on conversion rate [44–46]. To determine the apparent activation energy of these starches, the modified Coats-Redfern, FWO, model, and Kissinger-Akahira-Sunose (KAS) model were utilized.

### Theoretical approach of kinetics

The following is a generic description of the fundamental rate equation utilized in all kinetic studies:

1 $$\frac{{d \propto }}{{dt}}=kf~\left( \propto \right)$$

Equation (1) represents the rate of conversion $\frac{{d \propto }}{{~dt}}$, as a function of the rate constant and reactant concentration loss at a constant temperature. The conversion rate ∝ in this study is defined as follows:

2 $$ \propto =~\frac{{{W_0} - {W_t}}}{{{W_0} - {W_f}}}$$

where$~{W_t}$,$~{W_0}$, and ${W_f}$ are sample weight at time t, initial weight, and final weight, respectively. The rate constant k is generally given by the Arrhenius equation:

3 $$k=~A~exp\left( { - \frac{E}{{RT}}} \right)~$$

Where A is the pre-exponential factor (min_1), T is the absolute temperature (K), R is the gas constant (8.314 J/K mol), and E is the apparent activation energy (kJ/mol). Equations (1) and (3) when combined result in the relationship seen below:

4 $$\frac{{{\mathrm{d}} \propto }}{{{\mathrm{dt}}}}={\mathrm{A~exp}}\left( { - \frac{{\mathrm{E}}}{{{\mathrm{RT}}}}} \right){\mathrm{f~}}\left( \propto \right)$$

When a dynamic TGA technique is used,

4 $$\frac{{{\mathrm{d}} \propto }}{{{\mathrm{dT}}}}=\frac{{\mathrm{A}}}{{\mathrm{\boldsymbol{\upbeta}}}}{\mathrm{~exp}}\left( { - \frac{{\mathrm{E}}}{{{\mathrm{RT}}}}} \right){\mathrm{f~}}( \propto $$

Equations (4) and (5) represent the basic formulas for analytical techniques used to determine the kinetic parameters using TGA data. Table 1 provides a summary of activation energies for each model at different conversion levels. A number of models have been constructed based on these basic equations.$f~\left( \propto \right)$ Can be proposed in the following form,

6 $$f~\left( \propto \right)={\left( {1 - \propto } \right)^n}$$

The heating rate in the non-isothermal TGA studies is likewise a function of time and can be written as follows:

7 $$\frac{{d \propto }}{{dT}}=\frac{{d \propto }}{{dt}}~\frac{{dt}}{{dT}}~$$

8 $${\mathrm{\boldsymbol{\upbeta}}}={\mathrm{~}}\frac{{{\mathrm{dT}}}}{{{\mathrm{dt}}}}{\mathrm{~}}$$

9 $${\mathrm{ln}}\frac{\beta }{{{\mathrm{T}}_{{\mathrm{m}}}^{2}}}={\mathrm{ln}}\left( {\frac{{{\mathrm{AR}}}}{{{\mathrm{g}}\left( {\mathrm{x}} \right){{\mathrm{E}}_{\mathrm{a}}}}}} \right){\mathrm{~}} - \frac{{{{\mathrm{E}}_{\mathrm{a}}}}}{{{\mathrm{R}}{{\mathrm{T}}_{\mathrm{m}}}}}.$$

**Table 1:** Summary of activation energies for each model at different conversion levels

∝	Friedman equation	E, kJ/mol	FWO equation	E, kJ/mol	KAS eation	E, kJ/mol
0.1	y= − 18,056 x + 30.33	143	y = − 17397x + 34.728	129	y = − 16305x + 20.121	130
0.2	y= − 19,516 x + 32	157	y=− 19165x + 36.696	144	y = − 18033x + 22.019	141
0.3	y = − 20102x + 32.7	161	y =− 19661x + 36.717	149	y = − 18500x + 21.989	148
0.4	y = − 20073x + 32	163	y =− 20042x + 36.743	158	y = − 18860x + 21.979	152
0.5	y = − 19494x + 30.	158	y = − 20841x + 37.465	161	y = − 19639x + 22.668	161
0.6	y = − 19105x + 28.8	154	y = − 20426x + 36.122	158	y = 19204x + 21.29	156
0.7	y = − 19567x + 28.5	162	y =− 17343x + 30.406	132	y = − 16097x + 15.534	129
0.8	y = − 12,962 + 17	100	y =− 12682x + 22.299	92	y = − 11395x + 7.3631	90
0.9	y = − 9633.9x + 11	73	y = − 9369.2x + 16.62	71	y = − 8032x + 1.6074	67

Plotting ln (d$\propto$/dt) vs. 1/T yields (Ea/R) for a given value of a. This is conversional Friedman technique. The isoconversional F-W-O method is an integral method that yields (Ea/R) from the slope of the line plotted at a certain conversion rate when log b is plotted against 1/T. Sequences of straight lines with slopes (Ea/R) are obtained by plotting the left hand side at each heating rate against 1/T at that heating rate.

**Fig. 11:**
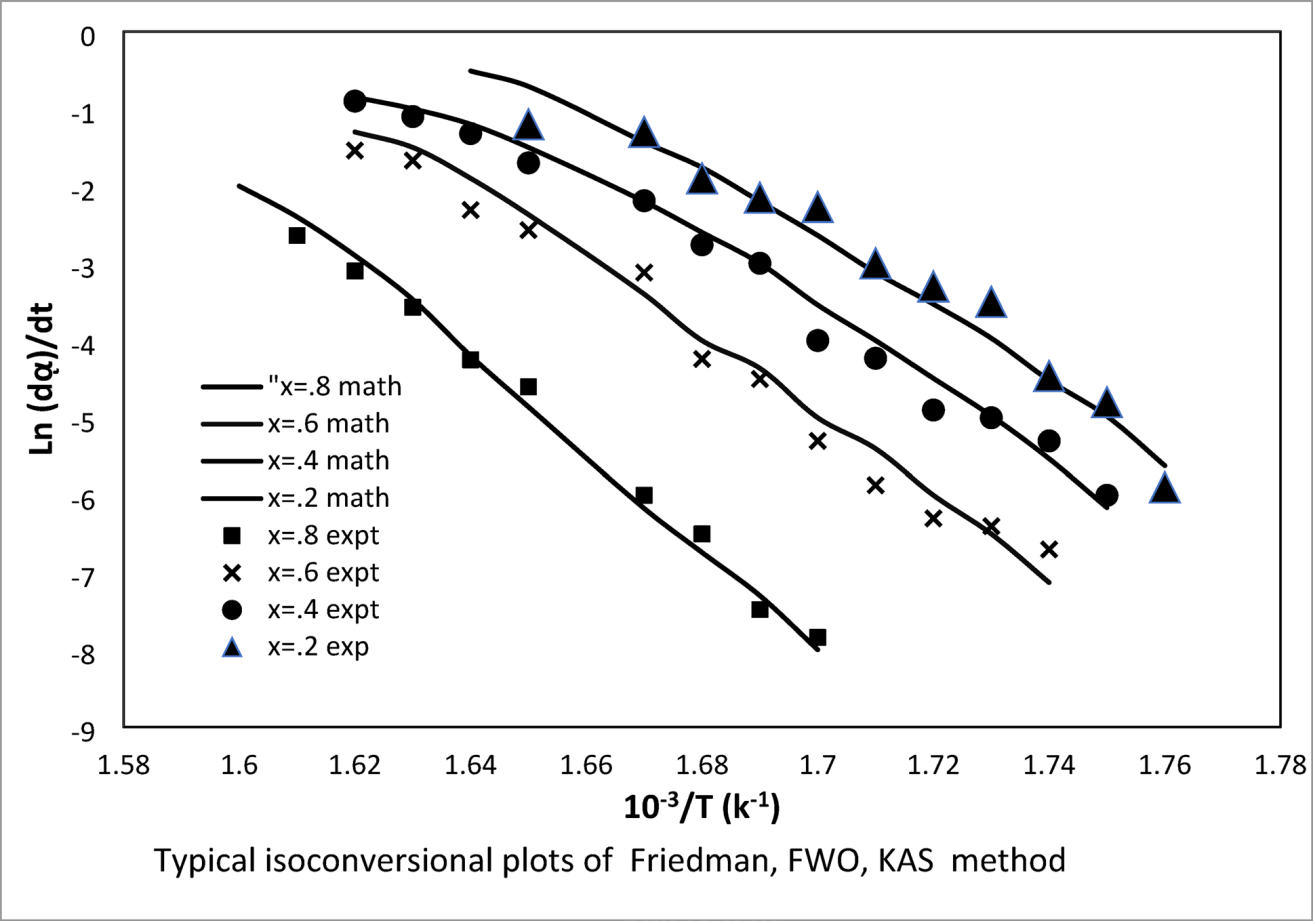
Regression coefficients (R^2^ values) for the first three reactions at the 1.5th level and the fourth reaction at the 1st order, along with average activation energies and their variations across different conversion levels

Figure 11 illustrates that the first three reactions had the highest regression coefficients at 1.5th level (R^2^ values: 0.9810, 0.9750, 0.9596), while the fourth reaction had the highest regression coefficient at 1st order (R^2^: 0.9737). Given that the lines in this study followed the same parallelism, the trend of the lines can be examined in three groups: ∝ = 0.1, 0.2–0.7, and 0.8–0.9. The average activation energies and the changes in activation energy for each model in response to varying conversions are displayed in Fig. 11. For every model in the specified order, the apparent activation energy was determined to be 143, 129, and 130 kJ/mol at a = 0.1. The computed activation energies for the interval ∝ = 0.2–0.7 were found to be within 157–161 kJ/mol, 144–161 kJ/mol, and 141–152 kJ/mol. The various kinetic methods including Kissinger, Friedman, FWO model and Kissinger-Akahira-Sunose, (KAS) model were used to determine the apparent activation energy of those starches. The results of activation energy show that the starch thermal decomposition has a multiple-step mechanism. The higher activation energy for high amylopectin starch could be explained by the higher molecular weight.

## Conclusions

This study investigated the properties of chitosan/starch composites with urea and thiourea as coupling agents and stearic acid as a plasticizer. Infrared (IR) spectroscopy revealed that urea-incorporated composites exhibited strong hydrogen bonding, while thiourea composites showed enhanced stability due to the presence of sulfur. X-ray diffraction (XRD) patterns confirmed these interactions, with urea-modified composites demonstrating better compatibility and stronger interactions, while thiourea composites displayed a more amorphous structure. SEM images supported these findings, showing good particle dispersion in the urea composite and partial compatibility with some aggregation in the thiourea composite. Dynamic mechanical analysis (DMA) indicated higher storage and loss moduli for the thiourea composite, reflecting superior mechanical properties and stability. Thermogravimetric Analysis (TGA) showed that the Chi/Str composite had enhanced thermal stability. The Chi/Str/Urea composite had a two-step degradation process, with a residual weight of about 23%, while the Chi/Str/Thio composite demonstrated the highest thermal stability accompanied by a residual weight of approximately 32% at 600 °C.

Kinetic analysis using the modified Coats-Redfern, F-W-O, and Kissinger models supported these findings, indicating higher thermal stability for the thiourea composite.

Overall, thiourea significantly enhances the mechanical and thermal properties of chitosan/starch composites, making them suitable for applications requiring improved durability and stability. Urea also improves properties but is less effective than thiourea. These results provide valuable insights for optimizing biopolymer composites for industrial applications.

## Data Availability

The datasets used and/or analyzed during the current study available from the corresponding author on reasonable request.
